# Shall the Dentist Advertise?

**Published:** 1898-03

**Authors:** William W. Belcher

**Affiliations:** Rochester, N. Y.


ARTICLE V.

### SHALL THE DENTIST ADVERTISE?

BY WILLIAM W. BELCHER, D. D. S., ROCHESTER, N. Y.

The question whether or not a professional man shall advertise is not a new one. In the medical profession the position of the man who advertises in the public prints is well understood, but there is one difficulty under which our sister profession has not labored. The medical man has nothing to sell but his services; if, perchance, the physician constructed artificial limbs, crutches or eye glasses, the question would be a more troublesome one.

In dentistry the mechanical and professional are so closely interwoven that there seems little chance of their separation.

It is nice to be independent, to do business on a side street or in the top story of a lofty office building, and say, "if the public desire my services they may come where I am"; but are we doing our whole duty to the public ?

In talking with some of the people who have patronized the dental parlors of our city, and who, after trying one quack have fallen into the hands of another, and finally come to me full of suspicion, with no confidence in mankind in general and dentists in particular, I have said to them: "There are dozens of men in this city who are giving honest and faithful services; why after you had one failure with the cheap man, did you not try one of them ?" 'Ah! but I am a comparative stranger and knew no dentists but those who advertise." What argument can be advanced to such a patient ?

The question of advertising is one that must be decided on entering practice and to the average college graduate the question of immediate gain, or future gain with respectability, are trying ones, indeed, I have been surprised to learn from men who were full of years and honors that they at times had been tempted to thrown down the gaunlet, enter the arena of low fees, fatten their bank account and clothe their family in fine linen.

The man who is seeking the road to wealth is misdirected when he chooses the dental profession. The average dentist lives well, spends freely, dies poor and in debt.

Should the young man just starting in practice advertise? I see no objection to his doing so in a professional manner. There are no such stricklers for the ethics of our profession as those to whom they have been but a bitter and grinding rule of conduct.

To have been heard of, if only once, is a great advantage with the new patient; the young man just commencing his career has a bitter realization of this fact, and while he is willing to conduct himself in a professional manner, feels the necessity of bringing his name before the public. He sincerely desires to live up to the code of ethics. But what would be advertising for him is ethical to the big fellow with a reputation who does not hesitate to see that his big cases are reported in full for the Sunday papers; at times they have been illustrated with wood cuts that could have come from no other source than the office of the operator.

Should the younger man desire to advertise his name and address in a select weekly, or church publication, which is not used by the quack, I can see no harm. Such advertising cannot be with any expectation of direct results; the effect is simply that the public becomes familiar with his calling, name and address; this is valuable to any man, whether running a grocery store or a dental office.

But stop with the name and address; do not even say "examinations free," and for heaven's sake keep out of the theater programmes and daily papers, for they are almos exclusively in the hands of the quacks, and for this reason the reputable man, feeling that there must be a dividing line between himself and the incompetent, refuses to advertise at all. I claim that if one city paper, of high standing, should make an effort to have a professional directory of medical and dental men, excluding the quacks and incompetents, society members exclusively, of accepted standing, each with name and address, it would be a good thing for the paper, the public and the profession.

Honor and glory are not nourishing, but to the true professional man dearer than wealth. Every man has two reputations; one among his professional brethren and one with the public. The first is most dear, but the latter brings his living and often we find a man with no professional standing who succeeds for a time in deluding the public, to the benefit of his exchequer. Many of us in the dental profession are woefully lacking in business principles, and perhaps the advertising dentist is better endowed than his fellows; but the advancement of dentistry has not come through the advertising offices; they are simply parasites, who live on the work of others, pulling them down by defrauding the public; they contribute nothing to the upbuilding or advancement of the profession or the investigation of new problems.

The man who advertises a "dental parlor," who decorates his office with valuable (?) oil paintings or advertises superiority over all his fellows, is to be avoided; likewise, the man who advertises cheapness, for if his services were worth more he could command the higher fee.

When we consider the preliminary educational requirements, the time and money expended, the standing of dentistry as a profession is not to be despised nor to be lightly traded for a mess of pottage by lowering it and your *alma mater* by resorting to the tricks and wiles of trade.

"Be good and you will be lonesome," says the humorous writer, but the only permanent foundation of a dental practice is honesty and ability. A loud tooting of trumpets and bragging in the daily papers will perhaps bring more immediate results, but not a desirable nor appreciative clientele, and will in the end be less profitable than a practice conducted on a dignified and professional basis.

There are many better ways of advertising—clean hands and linen, clean clothing, clean breath, clean instruments, and a clean office with modern instruments, a delicate touch, neat and tasty stationery, all are great advertisers, as are attendance and assuming a leading part in church and society work.

Every man should join his local dental society and take an active part. This is one of the best ways to advertise. No man is sufficient unto himself; if he be a superior dentist he owes it to his profession to join the society and demonstrate his superiority; if he feels himself deficient he should join the society that he make himself more proficient by exchanging ideas and methods with his fellows. It is not claimed that all the able men of the profession are society members; speed the day when this may be true; nor that every man who advertises in an unprofessional manner is incompetent, though this is true as a rule.

We have in our dental societies, men who compare well with their fellows in ability, honor and integrity, who have secured a practice by advertising with cuts of teeth and pictures, and after establishing a practice and not finding it necessary to continue advertising, apply to the society for membership.

What is your pleasure, gentlemen. Shall the man who has conducted himself in an unprofessional manner, join the societies on an equal footing with the young man who is just entering practice and trying to conduct himself in strict accord with the code of ethics? Will he not also conclude to advertise, and when he has secured a practice become a society member? If we expect him to follow the straight and narrow path, the reward of improper conduct should not be the too easy admittance in the society.

I am aware that the views herein expressed are not popular, but I believe they are based on common sense. "The name that dwells on every tongue no ministrel needs"; but there are other dentists who do not need the ministrel, and while there may be no good excuse for their being, they, too, must live.

